# Supplementary figures and images for: Immune profiling of Mycobacterium tuberculosis-specific T cells in recent and remote infection
Source: eBioMedicine. 2021 Feb 18;64:103233. doi: 10.1016/j.ebiom.2021.103233 (PMC7902886; doi:10.1016/j.ebiom.2021.103233)

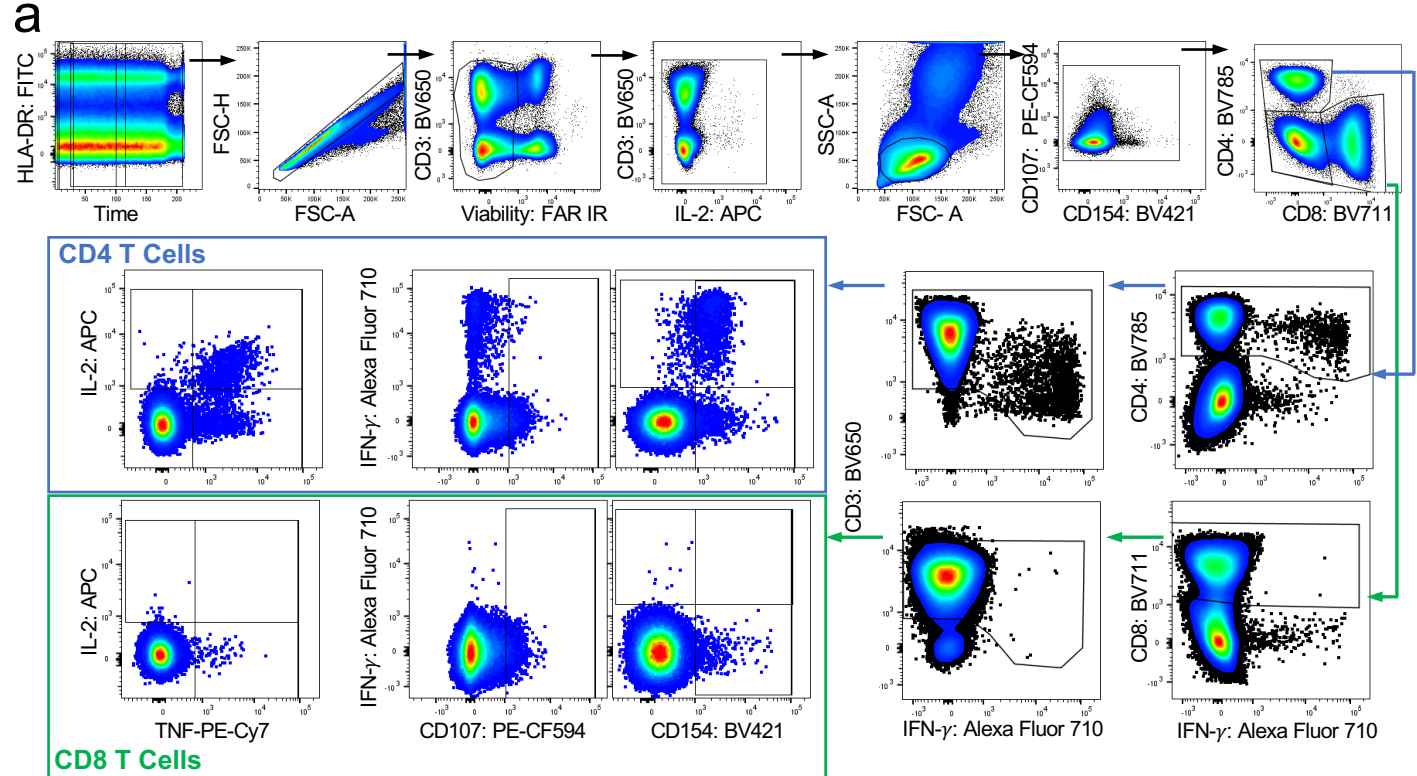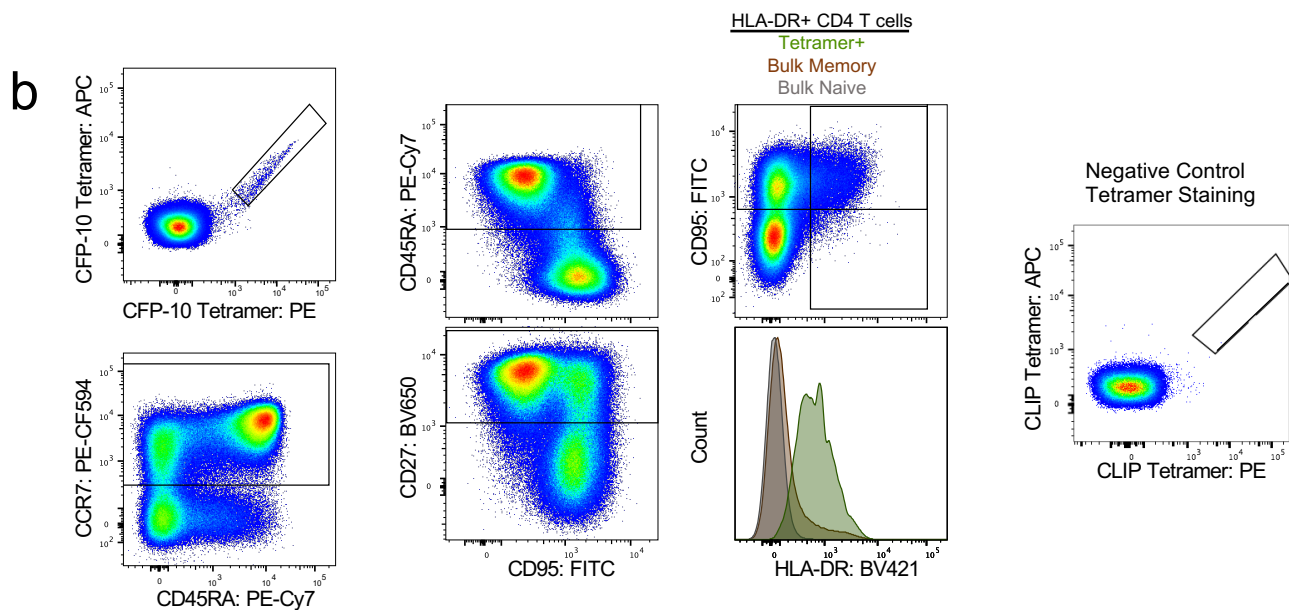

Supplement: Supplementary file 1 [file mmc1.pdf]

**a**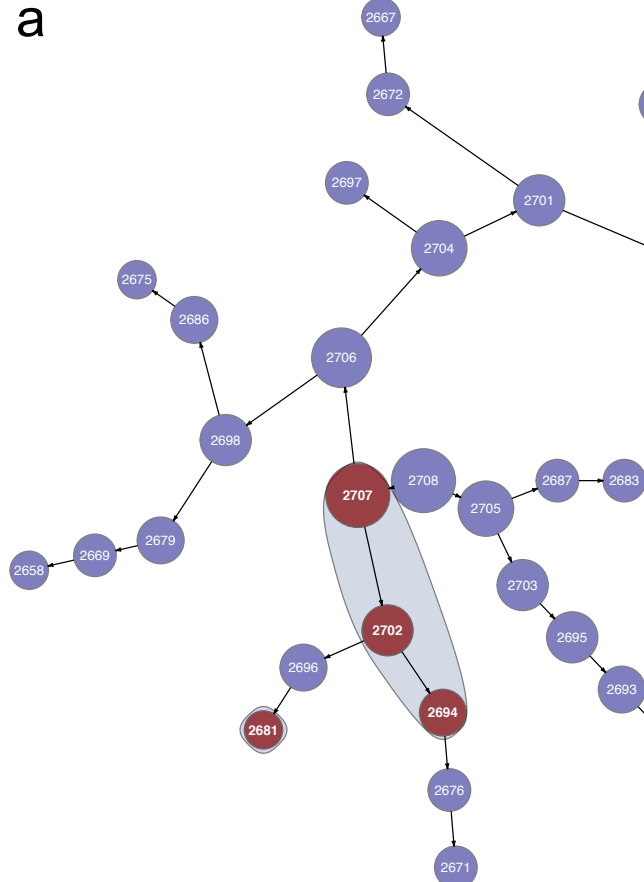**b**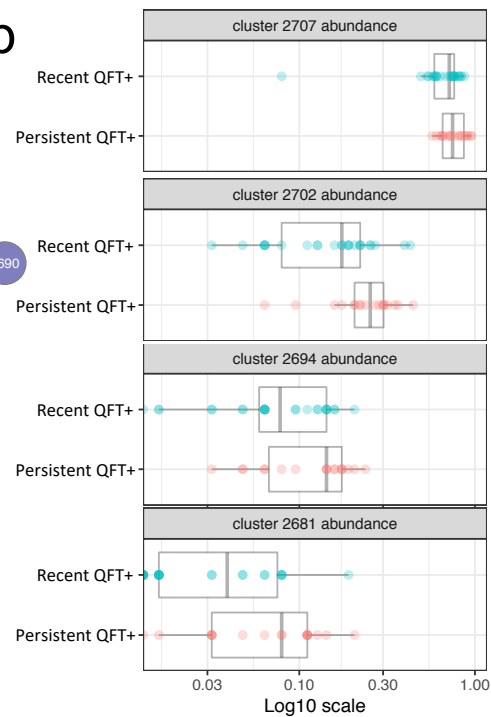**C**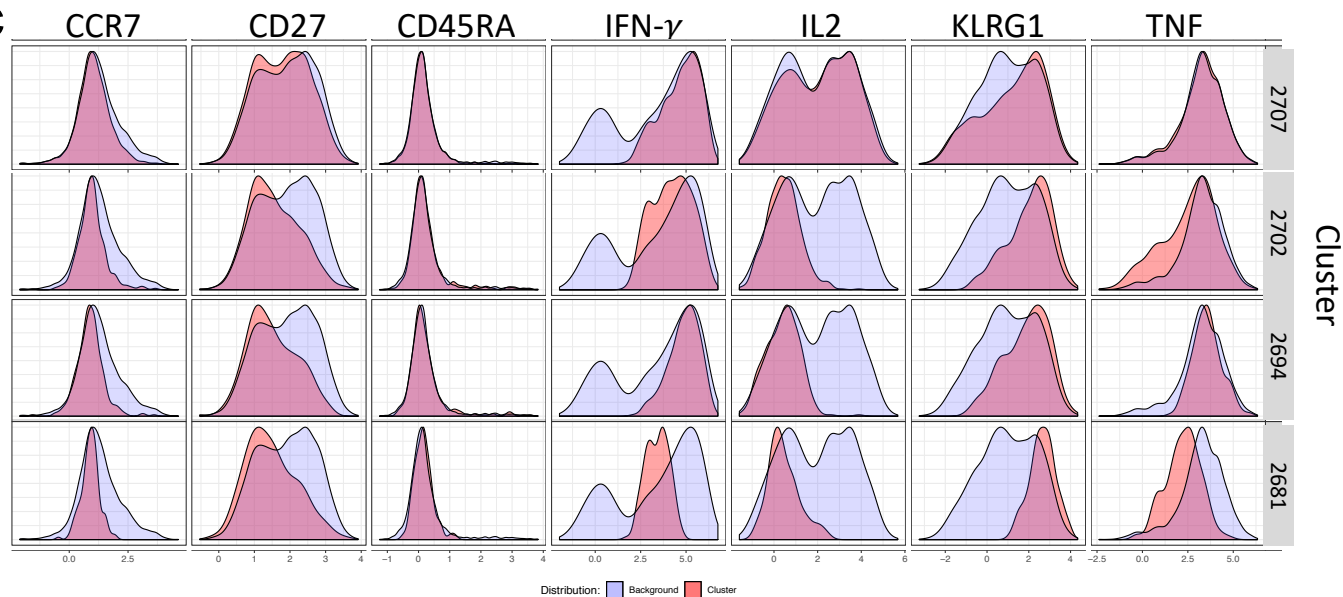

Supplement: Supplementary file 3 [file mmc3.pdf]

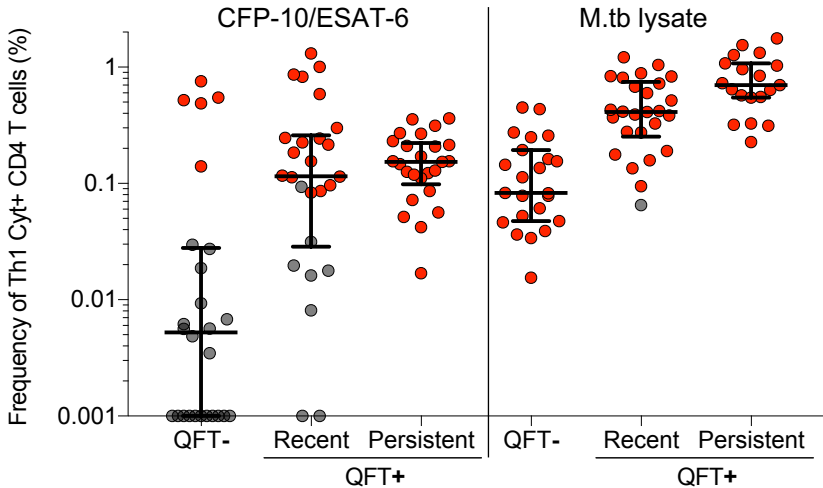

Supplement: Supplementary file 5 [file mmc5.pdf]

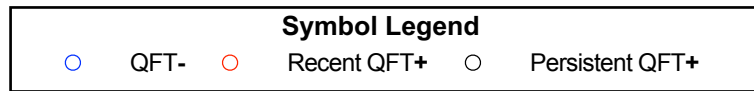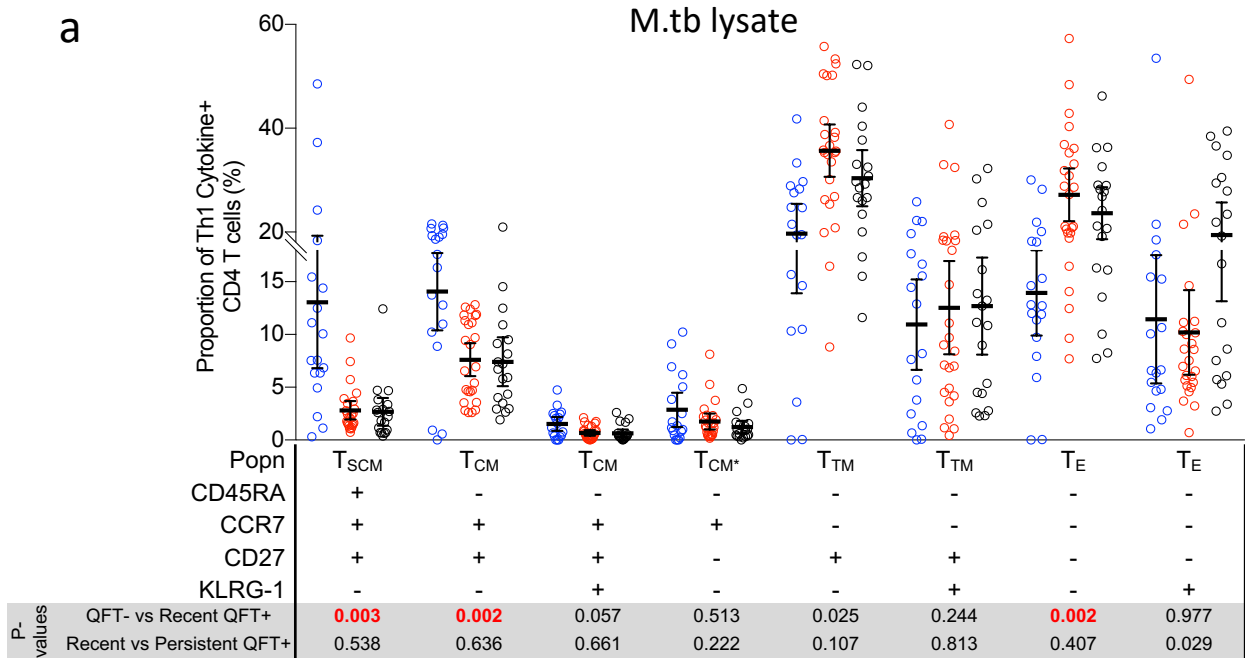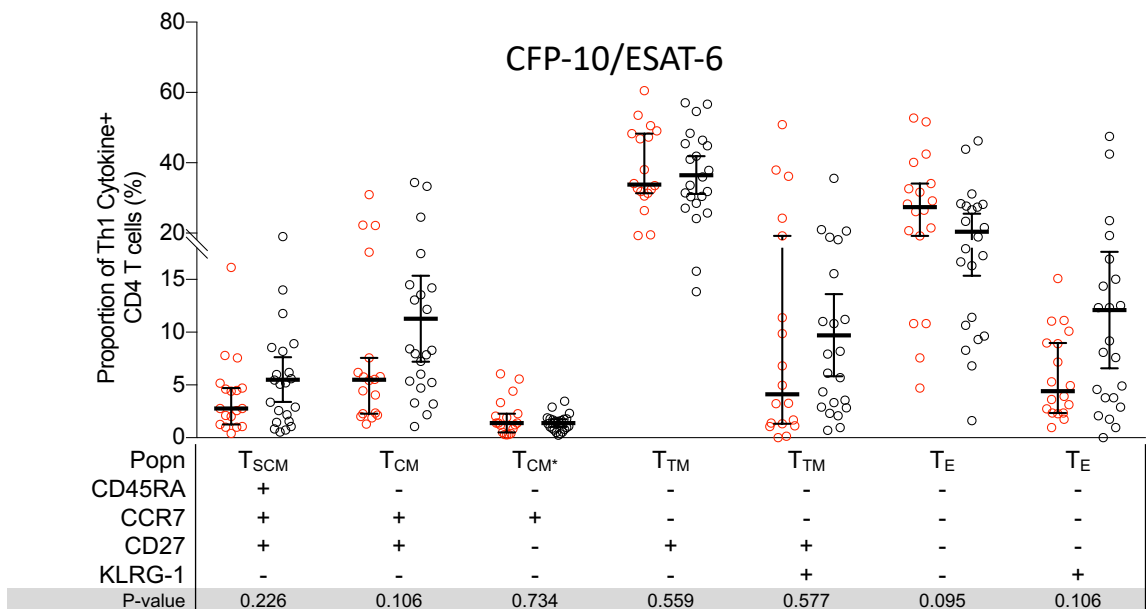

Supplement: Supplementary file 6 [file mmc6.pdf]
