# Supplementary material for: Immune profiling of Mycobacterium tuberculosis-specific T cells in recent and remote infection
Source: eBioMedicine. 2021 Feb 18;64:103233. doi: 10.1016/j.ebiom.2021.103233 (PMC7902886; doi:10.1016/j.ebiom.2021.103233)
Supplement: Supplementary file 2 [file mmc2.pdf]

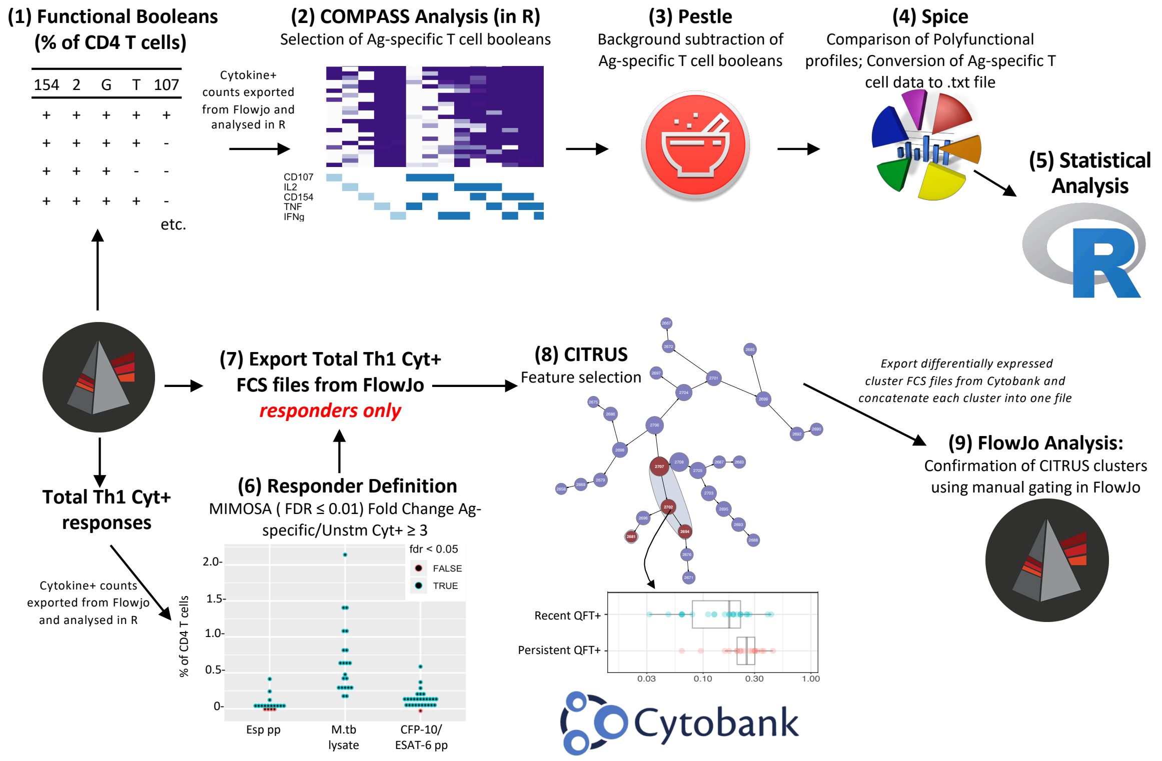
(1) Functional Booleans (% of CD4 T cells)
154 2 G T 107
+ + + + +
+ + + + -
+ + + - -
+ + + + -
etc.
Cytokine+ counts exported from Flowjo and analysed in R
(2) COMPASS Analysis (in R)
Selection of Ag-specific T cell booleans
CD107
IL2
CD154
TNF
IFNg
(3) Pestle
Background subtraction of Ag-specific T cell booleans
(4) Spice
Comparison of Polyfunctional profiles; Conversion of Ag-specific T cell data to .txt file
(5) Statistical Analysis
Total Th1 Cyt+ responses
Cytokine+ counts exported from Flowjo and analysed in R
(6) Responder Definition
MIMOSA (FDR ≤ 0.01) Fold Change Ag-specific/Unstm Cyt+ ≥ 3
fdr < 0.05
FALSE
TRUE
% of CD4 T cells
2.0-
1.5-
1.0-
0.5-
0-
Esp pp
M.tb lysate
CFP-10/ ESAT-6 pp
(7) Export Total Th1 Cyt+ FCS files from FlowJo
responders only
(8) CITRUS
Feature selection
Recent QFT+
Persistent QFT+
0.03
0.10
0.30
1.00
Cytobank
Export differentially expressed cluster FCS files from Cytobank and concatenate each cluster into one file
(9) FlowJo Analysis:
Confirmation of CITRUS clusters using manual gating in FlowJo
