# Supplementary material for: Immune profiling of Mycobacterium tuberculosis-specific T cells in recent and remote infection
Source: eBioMedicine. 2021 Feb 18;64:103233. doi: 10.1016/j.ebiom.2021.103233 (PMC7902886; doi:10.1016/j.ebiom.2021.103233)
Supplement: Supplementary file 4 [file mmc4.pdf]

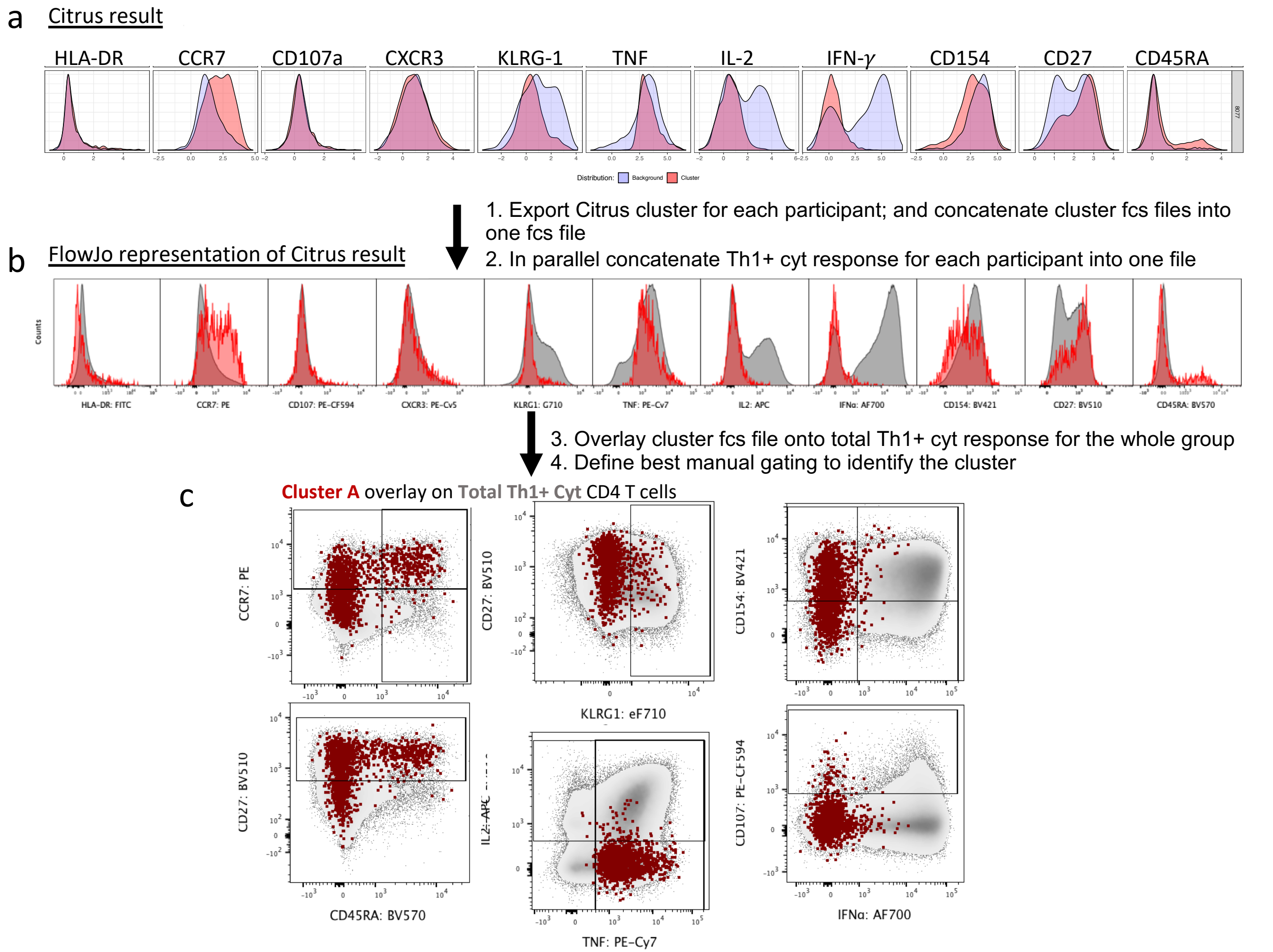
a
Citrus result
HLA-DR
CCR7
CD107a
CXCR3
KLRG-1
TNF
IL-2
IFN-γ
CD154
CD27
CD45RA
Distribution: Background Cluster
1. Export Citrus cluster for each participant; and concatenate cluster fcs files into one fcs file
2. In parallel concatenate Th1+ cyt response for each participant into one file
b
FlowJo representation of Citrus result
Counts
HLA-DR: FITC
CCR7: PE
CD107: PE-CF594
CXCR3: PE-Cy5
KLRG1: G710
TNF: PE-Cy7
IL2: APC
IFNa: AF700
CD154: BV421
CD27: BV510
CD45RA: BV570
3. Overlay cluster fcs file onto total Th1+ cyt response for the whole group
4. Define best manual gating to identify the cluster
c
Cluster A overlay on Total Th1+ Cyt CD4 T cells
CCR7: PE
CD27: BV510
CD154: BV421
KLRG1: eF710
CD27: BV510
CD45RA: BV570
IL2: APC
TNF: PE-Cy7
CD107: PE-CF594
IFNa: AF700
