# Supplementary material for: Immune profiling of Mycobacterium tuberculosis-specific T cells in recent and remote infection
Source: eBioMedicine. 2021 Feb 18;64:103233. doi: 10.1016/j.ebiom.2021.103233 (PMC7902886; doi:10.1016/j.ebiom.2021.103233)
Supplement: Supplementary file 7 [file mmc7.pdf]

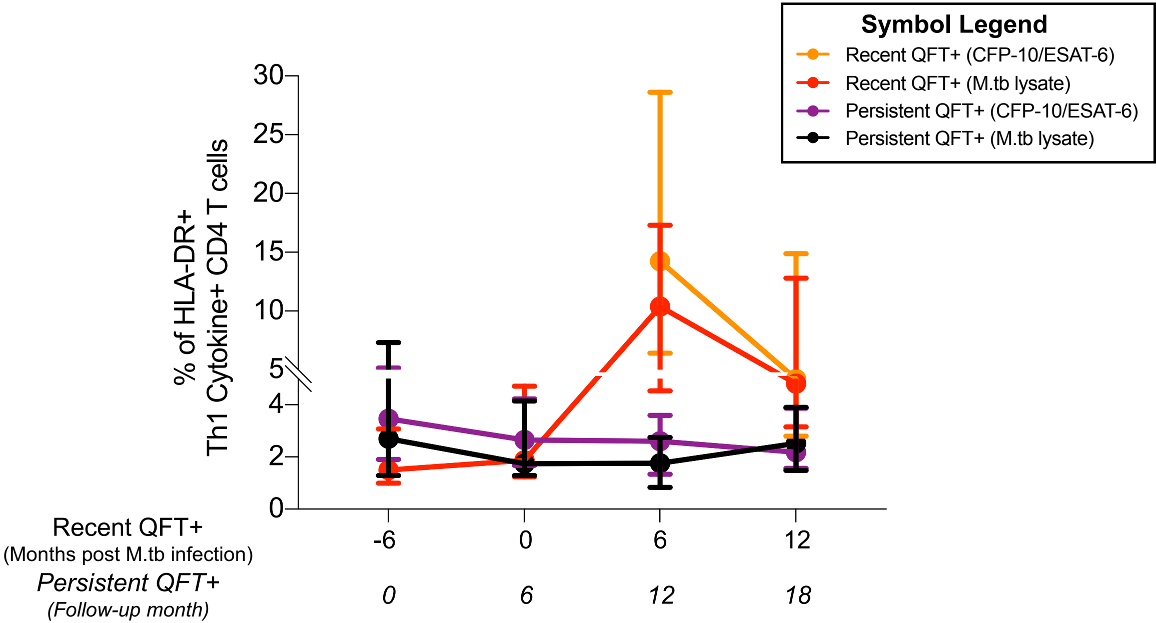

P-values			
	**Pair-wise comparison**	**CFP-10/ESAT-6**	**M.tb lysate**
Recent QFT+	-6 vs 0 months post M.tb infection	-	0.791
	0 vs +6 months post M.tb infection	-	**0.002**
	+6 vs +12 months post M.tb infection	**0.002**	0.064
Persistent QFT+	Follow up month 0 vs 6	0.225	0.009
	Follow up month 6 vs 12	0.169	0.120
	Follow up month 12 vs 18	0.829	0.946
