# Supplementary material for: Immune profiling of Mycobacterium tuberculosis-specific T cells in recent and remote infection
Source: eBioMedicine. 2021 Feb 18;64:103233. doi: 10.1016/j.ebiom.2021.103233 (PMC7902886; doi:10.1016/j.ebiom.2021.103233)
Supplement: Supplementary file 9 [file mmc9.docx]

ACS Study Team: Hassan Mahomed, Willem A. Hanekom, Fazlin Kafaar, Leslie Workman, Humphrey Mulenga, Rodney Ehrlich, Mzwandile Erasmus, Deborah Abrahams, Anthony Hawkridge, E. Jane Hughes, Sizulu Moyo, Sebastian Gelderbloem, Ashley Veldsman,

Michele Tameris, Hennie Geldenhuys, Gregory Hussey.

South African Tuberculosis Vaccine Initiative, Institute of Infectious Disease and Molecular Medicine, Division of Immunology, Department of Pathology, University of Cape Town, South Africa.
